# Supplementary material for: Linear algebraic structure of zero-determinant strategies in repeated games
Source: PLoS One. 2020 Apr 2;15(4):e0230973. doi: 10.1371/journal.pone.0230973 (PMC7117786; doi:10.1371/journal.pone.0230973)
Supplement: S1 Text — (PDF) [file pone.0230973.s001.pdf]

Supporting Information: Linear algebraic structure of zero-determinant strategies in repeated games

Masahiko Ueda[1,*] and Toshiyuki Tanaka[1,†]
[1]Department of Systems Science, Graduate School of Informatics, Kyoto University, Kyoto 606-8501, Japan

I. ZD STRATEGY IN ZERO-SUM GAMES

A. Absence of ZD strategies in the rock-paper-scissors game

We consider the rock-paper-scissors game:

$$\begin{aligned} \boldsymbol{s}_1 &= (0, 1, -1, -1, 0, 1, 1, -1, 0)^{\mathsf{T}} \\ \boldsymbol{s}_2 &= (0, -1, 1, 1, 0, -1, -1, 1, 0)^{\mathsf{T}} . \end{aligned} \tag{S1}$$

It should be noted that this game is two-player three-action symmetric zero-sum game. Because it is zero-sum game, the payoff vectors are linearly dependent $\boldsymbol{s}_2 = -\boldsymbol{s}_1$, and

$$\langle s_2 \rangle_{\mathrm{s}} \; = \; - \langle s_1 \rangle_{\mathrm{s}} \tag{S2}$$

always holds. When player 1 can employ ZD strategy, her strategy takes the form

$$\sum_{\sigma_1=1}^{3} c_{\sigma_1}^{(1)} \tilde{\boldsymbol{T}}_1(\sigma_1) \; = \; \alpha \boldsymbol{s}_1 + \gamma \boldsymbol{1}_9 \tag{S3}$$

and she enforces the linear relation

$$0 \; = \; \alpha \langle s_1 \rangle_{\mathrm{s}} + \gamma. \tag{S4}$$

Since the game is symmetric, player 2 can independently employ ZD strategy which enforces

$$0 \; = \; \alpha \langle s_2 \rangle_{\mathrm{s}} + \gamma. \tag{S5}$$

Then, because of the consistency of ZD strategies, $\gamma = 0$ must hold. On the other hand, by using $\gamma = 0$, Eq. (S3) can be written as

$$\alpha s_1 \left(\sigma_1^{\prime}, \sigma_2^{\prime} \right) \; = \; \sum_{\sigma_1=1}^{3} \left(c_{\sigma_1}^{(1)} - c_{\max}^{(1)} \right) \tilde{T}_1 \left(\sigma_1 | \sigma_1^{\prime}, \sigma_2^{\prime} \right) \qquad \left(\forall \sigma_1^{\prime}, \forall \sigma_2^{\prime} \right) \tag{S6}$$

$$= \; \sum_{\sigma_1=1}^{3} \left(c_{\sigma_1}^{(1)} - c_{\min}^{(1)} \right) \tilde{T}_1 \left(\sigma_1 | \sigma_1^{\prime}, \sigma_2^{\prime} \right) \qquad \left(\forall \sigma_1^{\prime}, \forall \sigma_2^{\prime} \right), \tag{S7}$$

where we have defined

$$c_{\max}^{(1)} \; \equiv \; \max_{\sigma_1} c_{\sigma_1}^{(1)} \tag{S8}$$

$$c_{\min}^{(1)} \; \equiv \; \min_{\sigma_1} c_{\sigma_1}^{(1)} \tag{S9}$$

$$\sigma_{1,\max} \; \equiv \; \arg\max_{\sigma_1} c_{\sigma_1}^{(1)} \tag{S10}$$

$$\sigma_{1,\min} \; \equiv \; \arg\min_{\sigma_1} c_{\sigma_1}^{(1)} \tag{S11}$$

---

*ueda.masahiko.5r@kyoto-u.ac.jp
†tt@i.kyoto-u.ac.jp

Then we find that

$$\alpha s_1\left(\sigma_{1,\max},\sigma_2'\right) = \sum_{\sigma_1=1}^{3}\left(c^{(1)}_{\sigma_1}-c^{(1)}_{\max}\right)\tilde{T}_1\left(\sigma_1|\sigma_{1,\max},\sigma_2'\right)\leq 0 \qquad \left(\forall\sigma_2'\right) \tag{S12}$$

and

$$\alpha s_1\left(\sigma_{1,\min},\sigma_2'\right) = \sum_{\sigma_1=1}^{3}\left(c^{(1)}_{\sigma_1}-c^{(1)}_{\min}\right)\tilde{T}_1\left(\sigma_1|\sigma_{1,\min},\sigma_2'\right)\geq 0 \qquad \left(\forall\sigma_2'\right). \tag{S13}$$

Because $\sigma_{1,\max}\in\{1,2,3\}$ and $\sigma_{1,\min}\in\{1,2,3\}$, Eq. (S12) and Eq. (S13) are inconsistent with the definition of the payoff (S1). Therefore, we conclude that ZD strategy does not exist in the rock-paper-scissors game.

B. Example of ZD strategy in two-player three-action symmetric zero-sum game

We next consider the following two-player three-action symmetric zero-sum game, which is the slightly modified version of the game in the main text:

$$\begin{aligned} \boldsymbol{s}_1 &= (0,r,0,-r,0,0,0,0,0)^{\mathsf{T}} \\ \boldsymbol{s}_2 &= (0,-r,0,r,0,0,0,0,0)^{\mathsf{T}}. \end{aligned} \tag{S14}$$

We remark that $\boldsymbol{s}_1$ and $\boldsymbol{s}_2$ are linearly dependent $\boldsymbol{s}_2=-\boldsymbol{s}_1$. We choose strategies of player 1 as

$$\begin{aligned} \boldsymbol{T}_1(1) &= (1,1-p,1,p',0,0,0,0,0)^{\mathsf{T}} \\ \boldsymbol{T}_1(2) &= (0,q,0,1-q',1,1,0,0,0)^{\mathsf{T}} \\ \boldsymbol{T}_1(3) &= (0,p-q,0,q'-p',0,0,1,1,1)^{\mathsf{T}} \end{aligned} \tag{S15}$$

with $0\leq p\leq 1$, $0\leq q\leq 1$, $0\leq p'\leq 1$, $0\leq q'\leq 1$, $q\leq p$, and $p'\leq q'$. Then we obtain

$$r\frac{q'-q}{p'q-pq'}\tilde{\boldsymbol{T}}_1(1)+r\frac{p'-p}{p'q-pq'}\tilde{\boldsymbol{T}}_1(2) = \boldsymbol{s}_1. \tag{S16}$$

Therefore, this strategy is ZD strategy which control the payoffs of both players as $\left\langle s_1\right\rangle_{\mathrm{s}}=\left\langle s_2\right\rangle_{\mathrm{s}}=0$. It should be noted that Eq. (S12) and Eq. (S13) are satisfied for this game.

II. ZD STRATEGY IN GAME WITH PUBLIC MONITORING

A. Two-player two-action game

As an example of ZD strategy for a repeated imperfect-monitoring game, we consider a two-player two-action symmetric game [1]. We assume $\tau\in\{1,2\}$ and the probability $W\left(\tau|\boldsymbol{\sigma}'\right)$ is given by

$$W\left(1|1,1\right) = \frac{1}{2}, \tag{S17}$$

$$W\left(1|1,2\right) = w, \tag{S18}$$

$$W\left(1|2,1\right) = 1-w, \tag{S19}$$

$$W\left(1|2,2\right) = \frac{1}{2}. \tag{S20}$$

This model is different from the noisy games studied by Hao et al. [2], in that they consider τ as noisy states, taking four values $\tau\in\{gg,gb,bg,bb\}$ corresponding to the four states in the iterated prisoner's dilemma game, whereas ours considers τ as taking only two values, representing winning/losing of player 1. The payoff vectors are given by $\boldsymbol{s}_1=(R,S,T,P)^{\mathsf{T}}$ and $\boldsymbol{s}_2=(R,T,S,P)^{\mathsf{T}}$. We consider equalizer strategy for player 1:

$$\tilde{\boldsymbol{T}}_1(1) = \beta\boldsymbol{s}_2+\gamma\boldsymbol{1}_4. \tag{S21}$$

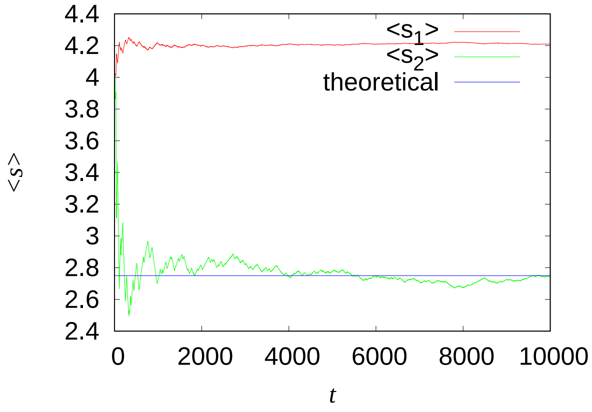

FIG. S1: Time-averaged payoffs of two players $\sum_{t'=1}^{t} s_n\left(\sigma_1(t'), \sigma_2(t')\right)/t$. The solid line corresponds to the theoretical prediction Eq. (S31) for player 2's expected payoff.

This strategy unilaterally sets the average payoff of player 2 in the steady state:

$$\langle s_2 \rangle_{\mathrm{s}} = -\frac{\gamma}{\beta}. \tag{S22}$$

By solving Eq. (S21) with respect to $\hat{T}_n\left(\sigma_n|\sigma'_n, \tau\right)$, we obtain

$$\hat{T}_1\left(1|1,1\right) = \frac{2(1-w)R-T}{1-2w}\beta+\gamma+1, \tag{S23}$$

$$\hat{T}_1\left(1|1,2\right) = \frac{T-2wR}{1-2w}\beta+\gamma+1, \tag{S24}$$

$$\hat{T}_1\left(1|2,1\right) = \frac{S-2wP}{1-2w}\beta+\gamma, \tag{S25}$$

$$\hat{T}_1\left(1|2,2\right) = \frac{2(1-w)P-S}{1-2w}\beta+\gamma. \tag{S26}$$

Concretely, we consider $w=1/5$ and $(R,S,T,P)=(4,1,9/2,3/2)$. By setting $\beta=-3/125$ and $\gamma=33/500$, we obtain

$$\hat{T}_1\left(1|1,1\right) = \frac{99}{100}, \tag{S27}$$

$$\hat{T}_1\left(1|1,2\right) = \frac{95}{100}, \tag{S28}$$

$$\hat{T}_1\left(1|2,1\right) = \frac{5}{100}, \tag{S29}$$

$$\hat{T}_1\left(1|2,2\right) = \frac{1}{100}, \tag{S30}$$

and

$$\langle s_2 \rangle_{\mathrm{s}} = \frac{11}{4}. \tag{S31}$$

In Fig. S1, we display the result of numerical simulation of one sample. Time-averaged payoffs of two players $\sum_{t'=1}^{t} s_n\left(\sigma_1(t'), \sigma_2(t')\right)/t$ are displayed when the strategy of player 2 is all-1:

$$\hat{T}_2\left(1|1,1\right) = 1 \tag{S32}$$

$$\hat{T}_2\left(1|1,2\right) = 1 \tag{S33}$$

$$\hat{T}_2\left(1|2,1\right) = 1 \tag{S34}$$

$$\hat{T}_2\left(1|2,2\right) = 1. \tag{S35}$$

The initial condition is set to $\sigma_1(0)=1$ and $\sigma_2(0)=1$. The numerical result for player 2 well matches with the theoretical prediction Eq. (S31). We can see that the expected payoff of player 2 is unilaterally controlled by the ZD strategy of player 1.

B. Two-player three-action game

We consider the same two-player three-action symmetric game in the main text:

$$\begin{aligned} \boldsymbol{s}_1 &= (0, r_1, 0, r_2, 0, 0, 0, 0, 0)^{\mathsf{T}} \\ \boldsymbol{s}_2 &= (0, r_2, 0, r_1, 0, 0, 0, 0, 0)^{\mathsf{T}} . \end{aligned} \tag{S36}$$

However, we assume that players can observe only common information $\tau \in \{\mathrm{y}, \mathrm{n}\}$ and the probability $W(\tau|\boldsymbol{\sigma}')$ is given by

$$W(\mathrm{y}|1,1) = 0 \tag{S37}$$
$$W(\mathrm{y}|1,2) = w \tag{S38}$$
$$W(\mathrm{y}|1,3) = 0 \tag{S39}$$
$$W(\mathrm{y}|2,1) = w \tag{S40}$$
$$W(\mathrm{y}|2,2) = 0 \tag{S41}$$
$$W(\mathrm{y}|2,3) = 0 \tag{S42}$$
$$W(\mathrm{y}|3,1) = 0 \tag{S43}$$
$$W(\mathrm{y}|3,2) = 0 \tag{S44}$$
$$W(\mathrm{y}|3,3) = 0. \tag{S45}$$

The common information τ represents whether payoffs of both players are non-zero or not. We consider the situation that player 1 employs the following strategy:

$$\hat{T}_1(1|1,\mathrm{y}) = \frac{w-p}{w} \tag{S46}$$
$$\hat{T}_1(1|1,\mathrm{n}) = 1 \tag{S47}$$
$$\hat{T}_1(1|2,\mathrm{y}) = \frac{p'}{w} \tag{S48}$$
$$\hat{T}_1(1|2,\mathrm{n}) = 0 \tag{S49}$$
$$\hat{T}_1(1|3,\mathrm{y}) = 0 \tag{S50}$$
$$\hat{T}_1(1|3,\mathrm{n}) = 0 \tag{S51}$$
$$\hat{T}_1(2|1,\mathrm{y}) = \frac{q}{w} \tag{S52}$$
$$\hat{T}_1(2|1,\mathrm{n}) = 0 \tag{S53}$$
$$\hat{T}_1(2|2,\mathrm{y}) = \frac{w-q'}{w} \tag{S54}$$
$$\hat{T}_1(2|2,\mathrm{n}) = 1 \tag{S55}$$
$$\hat{T}_1(2|3,\mathrm{y}) = 0 \tag{S56}$$
$$\hat{T}_1(2|3,\mathrm{n}) = 0 \tag{S57}$$
$$\hat{T}_1(3|1,\mathrm{y}) = \frac{p-q}{w} \tag{S58}$$
$$\hat{T}_1(3|1,\mathrm{n}) = 0 \tag{S59}$$
$$\hat{T}_1(3|2,\mathrm{y}) = \frac{q'-p'}{w} \tag{S60}$$
$$\hat{T}_1(3|2,\mathrm{n}) = 0 \tag{S61}$$
$$\hat{T}_1(3|3,\mathrm{y}) = 1 \tag{S62}$$
$$\hat{T}_1(3|3,\mathrm{n}) = 1 \tag{S63}$$

with $0 \leq p \leq w$, $0 \leq q \leq w$, $0 \leq p' \leq w$, $0 \leq q' \leq w$, $q \leq p$, and $p' \leq q'$. Then, from the definition

$$T_1(\sigma_1|\boldsymbol{\sigma}') \equiv \sum_{\tau=\mathrm{y},\mathrm{n}} W(\tau|\boldsymbol{\sigma}')\, \hat{T}_1(\sigma_1|\sigma'_1, \tau), \tag{S64}$$

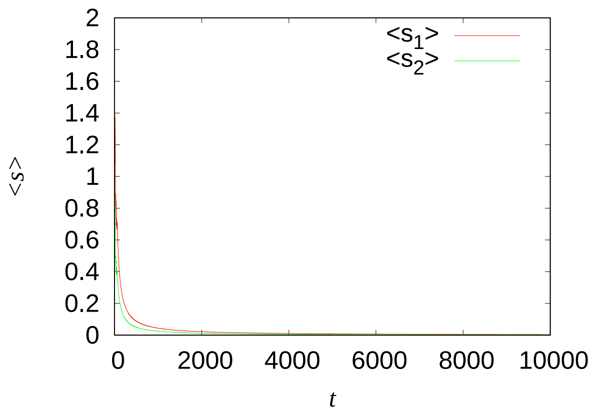

FIG. S2: Time-averaged payoffs of two players $\sum_{t'=1}^{t} s_n\left(\sigma_1(t'),\sigma_2(t')\right)/t$.

we obtain

$$\begin{aligned}
\boldsymbol{T}_1(1) &= \left(1,1-p,1,p',0,0,0,0,0\right)^{\mathsf{T}} \\
\boldsymbol{T}_1(2) &= \left(0,q,0,1-q',1,1,0,0,0\right)^{\mathsf{T}} \\
\boldsymbol{T}_1(3) &= \left(0,p-q,0,q'-p',0,0,1,1,1\right)^{\mathsf{T}}.
\end{aligned} \tag{S65}$$

These strategy vectors are the same as those in the main text, and give

$$\frac{q'r_1+qr_2}{p'q-pq'}\tilde{\boldsymbol{T}}_1(1)+\frac{p'r_1+pr_2}{p'q-pq'}\tilde{\boldsymbol{T}}_1(2) = \boldsymbol{s}_1 \tag{S66}$$

$$\frac{q'r_2+qr_1}{p'q-pq'}\tilde{\boldsymbol{T}}_1(1)+\frac{p'r_2+pr_1}{p'q-pq'}\tilde{\boldsymbol{T}}_1(2) = \boldsymbol{s}_2, \tag{S67}$$

which enforce the linear relations $\left\langle s_1\right\rangle_{\mathrm{s}}=0$ and $\left\langle s_2\right\rangle_{\mathrm{s}}=0$. Therefore, player 1 can enforce the same linear relations as those in the perfect monitoring case, even though players can know only τ. This means that the space of states Σ is successfully reduced to the smaller space $\{\mathrm{y},\mathrm{n}\}$ in terms of ZD strategies. The only difference is that the possible region of parameters p, q, p', q' is smaller for $w\neq 1$ than that in perfect monitoring case.

In Fig. S2, we display the result of numerical simulation of one sample for $r_1=2.0$ and $r_2=1.0$. Time-averaged payoffs of two players $\sum_{t'=1}^{t} s_n\left(\sigma_1(t'),\sigma_2(t')\right)/t$ are displayed when $w=0.9$, $p=0.2$, $q=0.1$, $p'=0.25$, $q'=0.3$ and the strategy of player 2 is

$$\hat{T}_2\left(\sigma_2|\sigma_2',\tau\right) = \frac{1}{3} \qquad \left(\forall\sigma_2,\forall\sigma_2',\forall\tau\right). \tag{S68}$$

The initial condition is given by the probability distribution $P(\sigma)=1/3$ for both players. The numerical result is consistent with the theoretical prediction $\left\langle s_1\right\rangle_{\mathrm{s}}=\left\langle s_2\right\rangle_{\mathrm{s}}=0$.

[1] R. Kobayashi, Master's thesis, Kyoto University (2018).

[2] D. Hao, Z. Rong, and T. Zhou, Phys. Rev. E **91**, 052803 (2015), URL `https://link.aps.org/doi/10.1103/PhysRevE.91.052803`.
